# Unusual site, familiar pathology: A case of fibroadenoma in ectopic breast tissue

**DOI:** 10.1016/j.radcr.2025.08.057

**Published:** 2025-09-11

**Authors:** Richard B. Schonour, Margaret H. Mowry

**Affiliations:** a Frederick P. Whiddon College of Medicine**,** University of South Alabama, 5795 USA Drive North, CSAB 170, Mobile, AL 36688, USA; b Department of Radiology, USA Health, 2451 University Hospital Drive, Mastin Building, Room 301, Mobile, AL 36617, USA

**Keywords:** Axillary fibroadenoma, Axillary mass, Fibroadenoma, Core needle biopsy, Ectopic breast tissue

## Abstract

Fibroadenomas are among the most common benign breast tumors, typically occurring within the breast parenchyma. However, their occurrence in the axilla is rare and often originates from ectopic breast tissue (EBT), which develops due to incomplete regression of the embryologic mammary ridge. Although EBT is present in up to 6% of women, it is usually asymptomatic. When a mass is detected in the axilla, particularly in younger women, it often raises concern for lymphadenopathy or malignancy. This overlap in clinical presentation creates a diagnostic challenge. A 35-year-old woman presented with a 6-month history of a painless, palpable mass in the right axilla. Physical examination revealed a soft, mobile nodule. Mammography and ultrasound classified the lesion as BI-RADS-4A. An additional retro-areolar mass in the left breast showed similar BI-RADS-4A features. Core needle biopsies from both sites confirmed fibroadenomas. Due to patient preference, the axillary mass was surgically excised, and recovery was uneventful. This case illustrates a rare presentation of fibroadenoma arising in axillary EBT, underscoring the importance of considering ectopic breast tissue in the differential diagnosis of axillary masses. Careful imaging evaluation is essential to distinguish benign lesions, which often appear as well-defined, oval, hypoechoic masses, from suspicious features like irregular margins or increased vascularity that may suggest malignancy. Increased awareness of this entity can reduce diagnostic uncertainty, prevent unnecessary interventions, and guide appropriate patient-centered management.

## Introduction

Fibroadenomas are the most common benign breast tumors in women of reproductive age [1]. They usually present as painless, well-circumscribed masses within the breast parenchyma. While fibroadenomas are familiar entities in clinical practice, their development in ectopic breast tissue, most commonly located in the axilla, is considerably less common and may present a diagnostic challenge. Ectopic breast tissue arises from incomplete regression of the embryologic mammary ridge [2] and can develop the same benign and malignant conditions as normally positioned breast tissue.

When a patient presents with an axillary mass, clinicians must consider a broad differential diagnosis that includes lymphadenopathy, lipomas, and primary breast malignancies. Careful evaluation with imaging, histopathologic confirmation, and consideration of patient symptoms guide diagnosis and management. This case report highlights the importance of recognizing ectopic breast tissue as a potential source of pathology and illustrates a systematic approach to the diagnostic workup of a BI-RADS-4A axillary lesion ultimately found to be a fibroadenoma.

## Case report

### Patient information

A 35-year-old female with a past medical history of hypertension presented to the clinic with a 6-month history of a palpable right axillary lump. She denied nipple discharge, breast tenderness, or skin changes.

### Physical exam

Physical examination revealed a soft, mobile nodule in the right axilla. The remainder of the exam was unremarkable.

### Diagnostic mammography

**Fig. 1:**
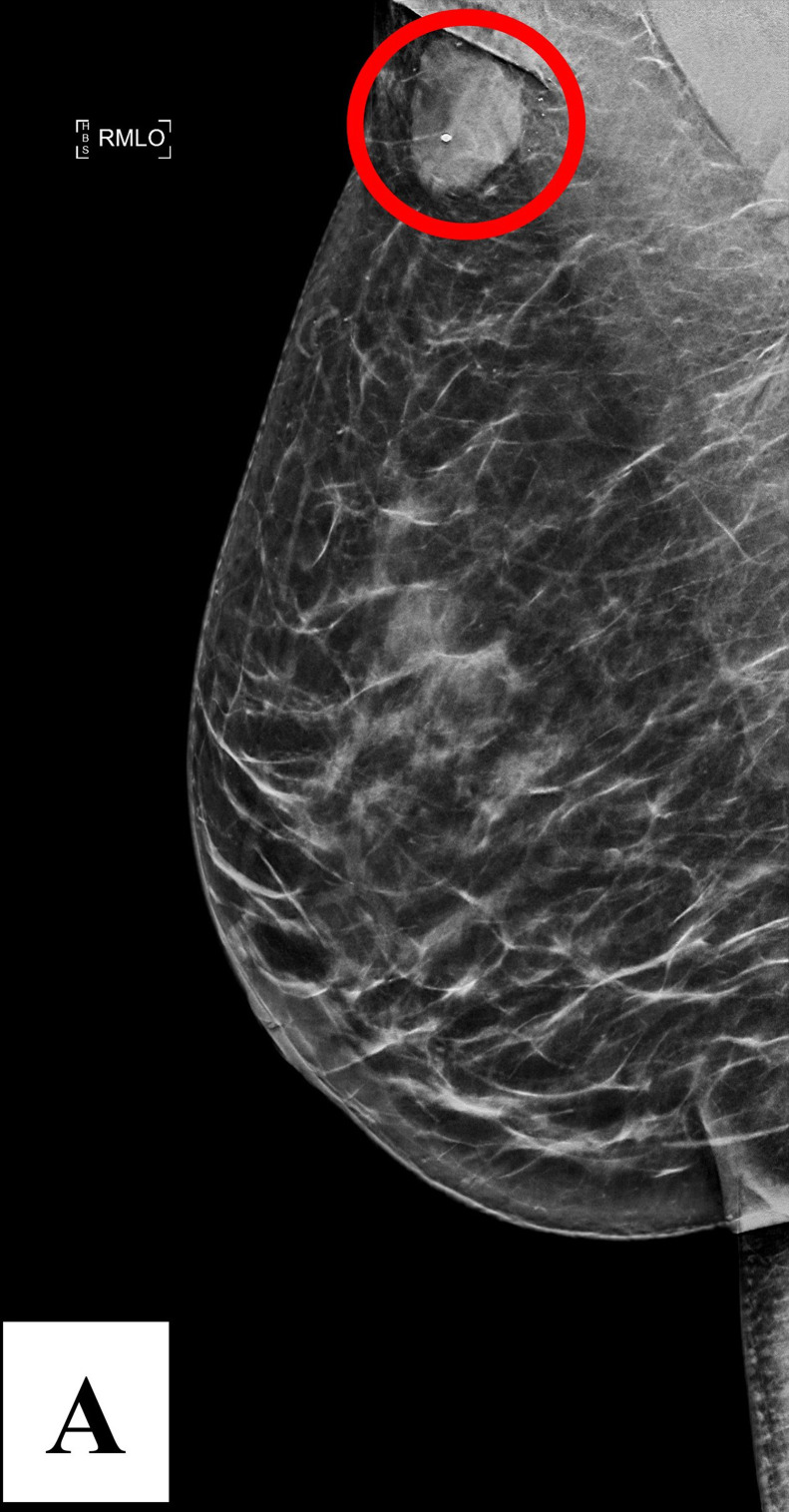
Diagnostic mammography (MLO C-view) demonstrates the presence of a 2.4 × 1.9 cm round, circumscribed, high-density mass in the right axillary tail (circled, A).

**Fig. 2:**
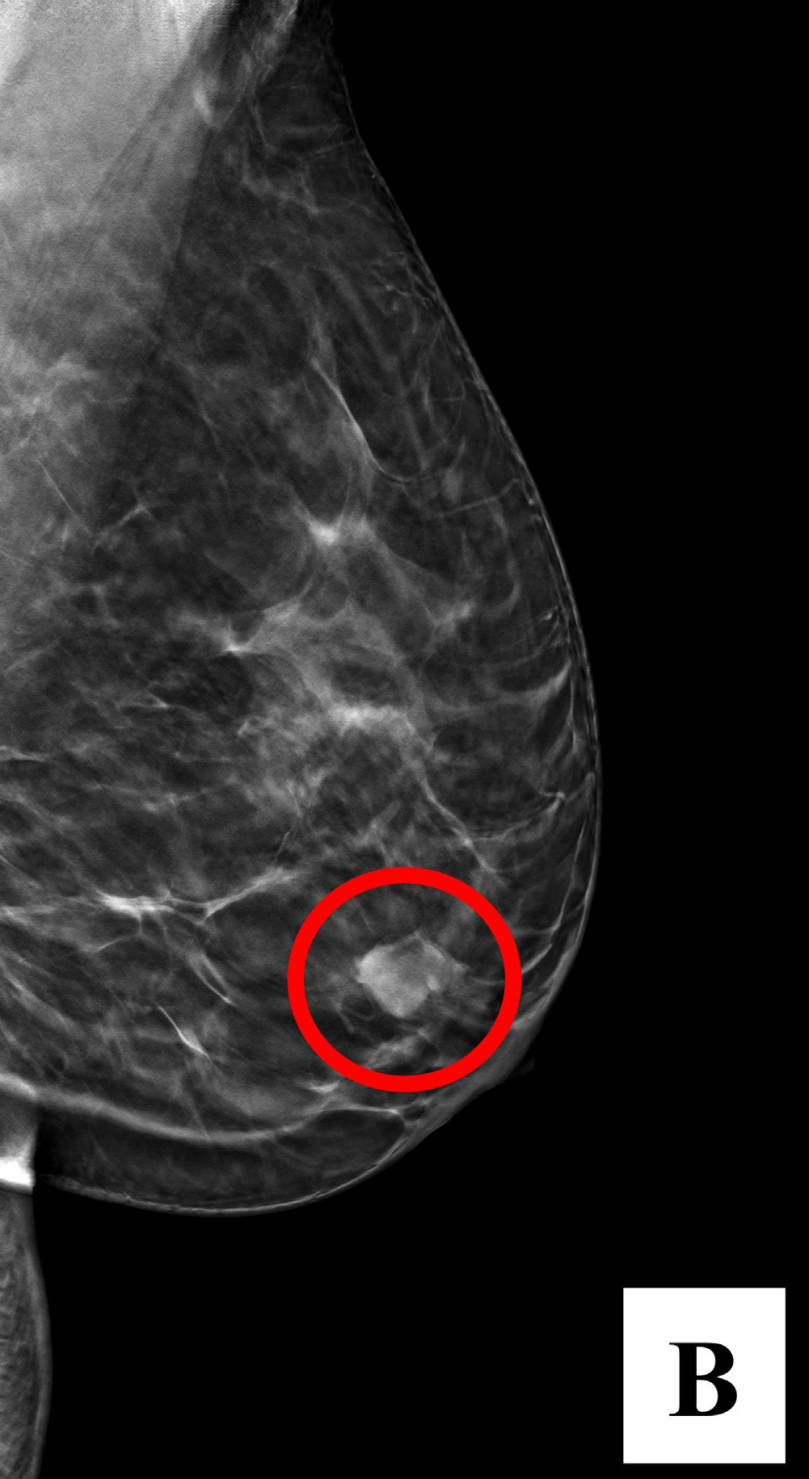
Diagnostic mammography (MLO C-view) incidentally found a 1.0 × 1.0 cm round, microlobulated, high-density mass in the left retro-areolar region (circled, B).

To start the clinical workup, the clinician ordered diagnostic imaging to further evaluate the mass. Bilateral diagnostic mammography demonstrated scattered areas of fibroglandular density. The mammogram identified a round, circumscribed, high-density mass measuring 2.4 × 1.9 cm in the right axillary tail (Fig. 1). The mammogram also noted a round, microlobulated, high-density mass measuring 1.0 × 1.0 cm in the left retroareolar region (Fig. 2). No malignant calcifications, architectural distortion, skin or nipple changes, or axillary adenopathy were observed in either breast.

### Ultrasound evaluation

**Fig. 3:**
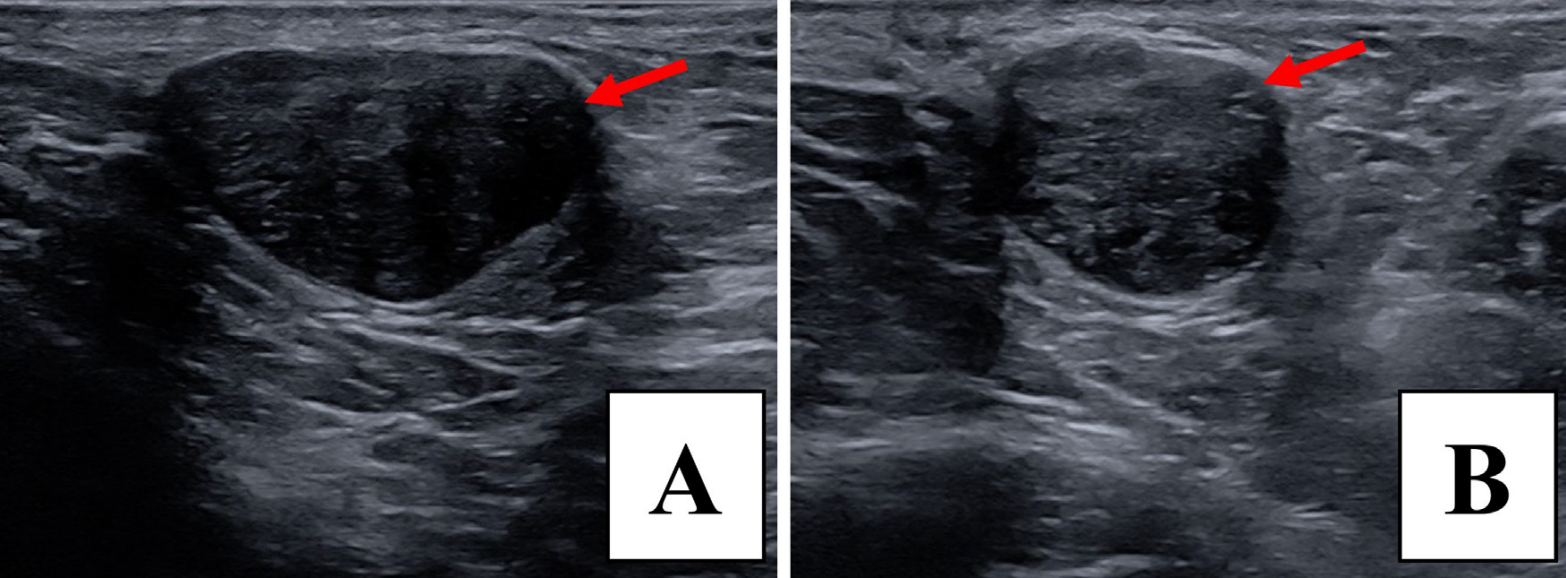
Ultrasound imaging demonstrates an oval, circumscribed, hypoechoic 2.8 × 1.6 × 1.9 cm mass in the right axilla in sagittal (A) and transverse (B) views, with red arrows highlighting the lesion.

**Fig. 4:**
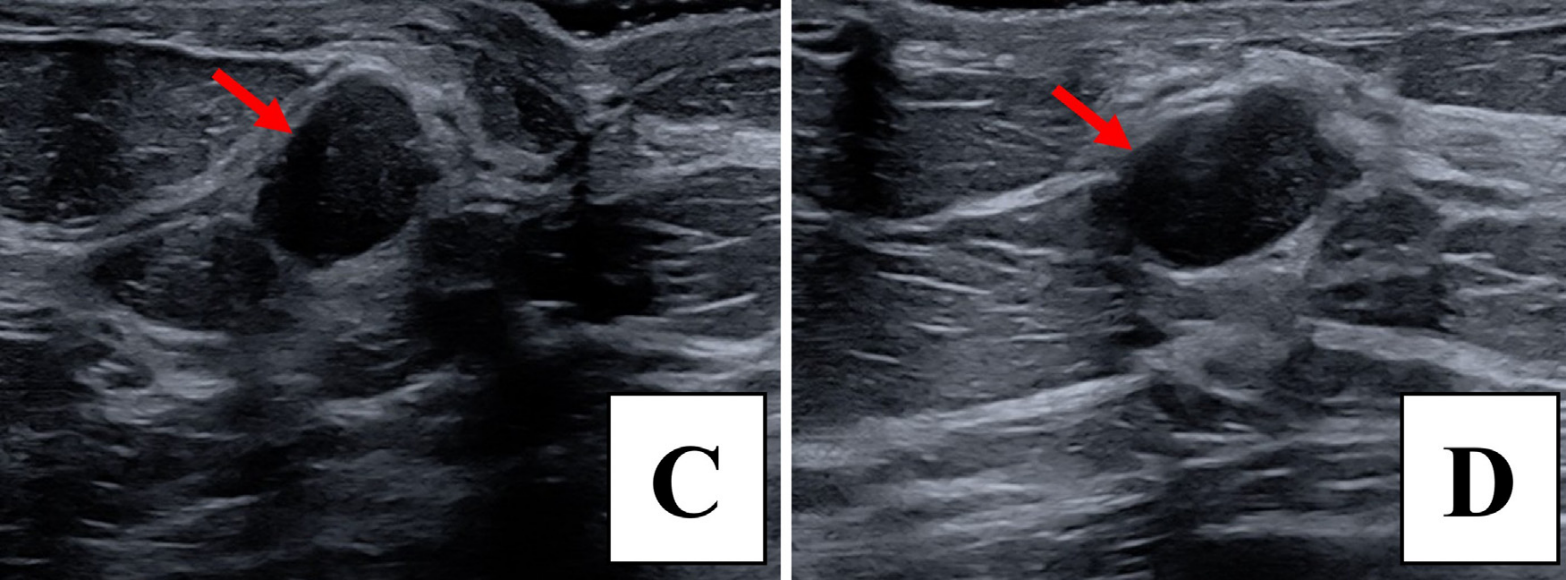
In the left retro-areolar region, ultrasound reveals an oval, parallel, hypoechoic 1.6 × 1.2 × 1.0 cm mass with angular margins in sagittal (C) and transverse (D) views, with red arrows highlighting the lesion.

On the same day, the radiology team performed bilateral breast ultrasound to better evaluate the internal characteristics of the mass. On the right, an oval, circumscribed, hypoechoic mass measuring 2.8 × 1.6 × 1.9 cm was visualized in the axillary region (Fig. 3). On the left, an oval, parallel, hypoechoic mass with angular margins measuring 1.6 × 1.2 × 1.0 cm was identified in the retroareolar region (Fig. 4).

### Correlation of findings

**Fig. 5:**
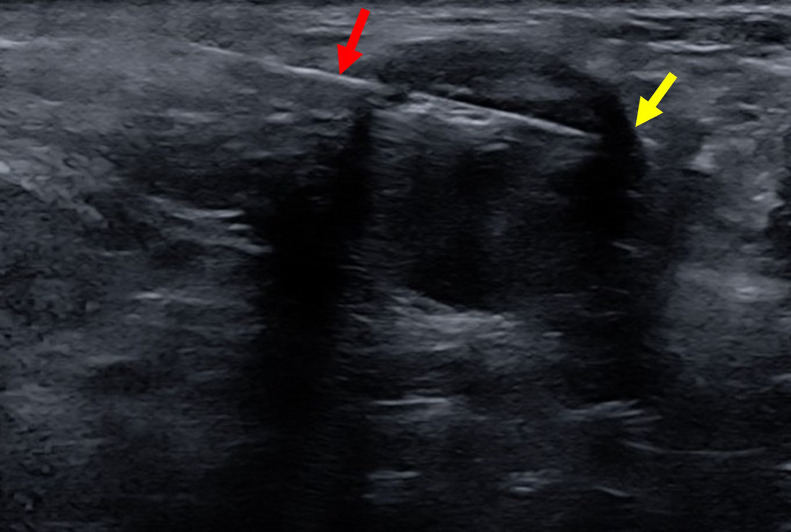
Transverse ultrasound image obtained during ultrasound-guided core needle biopsy of the right axillary lesion (yellow arrow). The 12-gauge biopsy guide (red arrow) was advanced into the lesion and 3 cores were obtained.

Ultrasound findings correlated with the masses seen on mammography. Both the right and left sided lesions were classified as BI-RADS-4A, with the right sided lesion’s designation driven primarily by its location in ectopic breast tissue. This classification indicated suspicious abnormalities, with an estimated risk of malignancy between >2% and ≤10%, and therefore warranted core needle biopsy. The radiologist performed ultrasound-guided core needle biopsies at both sites (Fig. 5). The pathology team confirmed fibroadenomas in both the right axilla and the left breast.

### Intervention and outcome

Given the symptomatic nature of the right axillary mass, the patient underwent an outpatient excisional biopsy. We managed the left-sided fibroadenoma conservatively with observation, given its smaller size and lack of symptoms.

## Discussion

Fibroadenomas are among the most common benign breast tumors, occurring in approximately 25% of women, typically between the ages of 15 and 35 [3,4]. While they most often develop within the breast, fibroadenomas can also arise in extra-mammary locations such as the axilla and face [5]. Their presence in the axilla is rare, with fewer than 40 cases [6,5]. reported in the literature (Table 1). When found in the axilla, these tumors originate from ectopic breast tissue (EBT), which results from incomplete regression of the embryologic mammary ridge. EBT is found in up to 6% [7]. of women but is frequently asymptomatic and may be mistaken for other soft tissue structures or pathologies [8].

**Table 1:** Summary of selected case reports highlighting fibroadenomas in ectopic breast tissue.

Author (Year)	Patient age	Presentation	Mass location	Imaging features	Management	Outcome
Surd et al. [5]	17	4 × 3 × 2.5-cm right axillary mass.	Right Axilla	Ultrasound scan revealed a homogenous 3 × 3 cm hypoechoic mass.	Surgical excision	Pathological diagnosis of fibroadenoma.
Coras et al. [9]	23	Reddish nodule in the right axilla measuring 2 × 2 cm.	Right Axilla	N/A	Initially thought to be an abscess, so attempted drained. No decrease in size, so excised under anesthesia.	Pathological diagnosis of fibroadenoma.
Gentile et al. [10]	58	5 × 6.5-cm subcutaneous mass in the left axillary region and a 5.5 × 6.0-cm mass in the right axillary region.	Bilateral Axillae	Clearly circumscribed homogeneous solid masses.	Surgical Excision	Pathological diagnosis of fibroadenoma.
Tee et al. [11]	20′s	2 Year history of progressive right axillary swelling.	Right Axilla	Well-circumscribed oval hypoechoic lesion measuring 3.1 cm × 1.4 cm × 2.7 cm.	Excisional biopsy due to patient concern.	Pathological diagnosis of fibroadenoma.

When evaluating an axillary mass, a broad differential diagnosis must be considered. Benign causes can include fibroadenomas arising from EBT, reactive or infectious lymphadenopathy, lipomas, sebaceous cysts, and hidradenitis suppurativa [12]. Malignant etiologies could include metastatic breast carcinoma to axillary lymph nodes, metastatic carcinoma from a nonbreast primary site, lymphoma, or a primary soft tissue sarcoma. Differentiating these pathologies depends on a combination of clinical history, physical exam, and imaging findings [12].

Evaluation begins with imaging, guided by the patient’s age and clinical presentation. In women over 30, diagnostic mammography is typically the first-line modality to assess for masses, calcifications, and other architectural abnormalities. If a palpable mass is present but not well visualized on mammogram, targeted ultrasound is performed to better characterize the lesion. Fibroadenomas typically appear as well-circumscribed, oval, hypoechoic masses with posterior acoustic enhancement [13]. In contrast, features suggestive of malignancy include irregular margins, heterogeneous echotexture, and increased internal vascularity on Doppler imaging [8].

Radiologists categorize imaging findings using the BI-RADS (Breast Imaging Reporting and Data System), which guides next steps in management. BI-RADS 1-2 indicates benign findings and typically warrants routine screening. BI-RADS 3 lesions are “probably benign”, often prompting short-term imaging follow-up (eg, in 6 months). BI-RADS 4, as in our case, reflects a “suspicious abnormality” and warrants tissue diagnosis by core needle biopsy [14].

While most fibroadenomas can be monitored safely with routine imaging follow-up, the decision to pursue surgical excision versus conservative management should be individualized based on several factors. Lesions that are symptomatic, such as those causing pain or with notable growth, often prompt consideration of excision to provide symptom relief [15]. Additionally, patient anxiety regarding the mass or its potential for malignancy can influence the choice toward surgical removal. This emphasizes the importance of patient-centered care and shared decision-making.

Imaging findings also play a critical role in guiding management. Lesions with atypical or suspicious features on ultrasound or mammography generally warrant tissue diagnosis by core needle biopsy. When biopsy confirms a benign diagnosis but clinical or imaging concerns persist, excisional biopsy may be preferred to definitively exclude malignancy and provide reassurance [16].

Overall, this case illustrates a rare presentation of a symptomatic axillary fibroadenoma arising from ectopic breast tissue, ultimately confirmed through biopsy and surgical excision. It underscores the importance of considering benign etiologies in the differential diagnosis of axillary masses, particularly in reproductive-age women. Awareness of ectopic breast tissue pathology and a stepwise, risk-stratified approach using imaging and BI-RADS classification are key to guiding accurate diagnosis and appropriate management.

## Conclusion

Axillary fibroadenoma, though uncommon, should be considered in the differential diagnosis of axillary masses, especially in young women. Greater awareness of ectopic breast tissue and its associated conditions can support accurate diagnosis and appropriate clinical decision-making.

## Availability of data and materials

All relevant data are presented within the manuscript and are available from the corresponding author upon reasonable request.

## Declaration of generative AI and AI-assisted technologies in the writing process

During the preparation of this work, the authors used OpenAI’s ChatGPT in order to improve language and readability. After using this tool, the authors reviewed and edited the content as needed and take full responsibility for the content of the publication.

## Patient consent

Written informed consent was obtained from the patient for the publication of this case report. The authors affirm that no personal identifiers are included and that all information has been sufficiently anonymized to protect patient confidentiality. In accordance with the University’s Institutional Review Board (IRB) Standard Operating Procedure 805 on case reports, this project did not require IRB review or approval.
